# A novel frameshift mutation in *DNAH6* associated with male infertility and asthenoteratozoospermia

**DOI:** 10.3389/fendo.2023.1122004

**Published:** 2023-06-22

**Authors:** Fei Huang, Jun Zeng, Dan Liu, Jing Zhang, Boluo Liang, Jingping Gao, Rong Yan, Xiaobo Shi, Jianlin Chen, Wanjuan Song, Hua-Lin Huang

**Affiliations:** Reproductive Medicine Center, Department of Obstetrics and Gynecology, The Second Xiangya Hospital, Central South University, Changsha, China

**Keywords:** infertility, asthenoteratozoospermia, sperm flagella, *DNAH6*, mutation, premature termination codon

## Abstract

**Introduction:**

Asthenoteratozoospermia is one of the most common causes of male infertility. Several genes have been identified as genetic causative factors, but there is a considerable genetic heterogeneity underlying asthenoteratozoospermia. In this study, we performed a genetic analysis of two brothers from a consanguineous Uighur family in China to identify gene mutations causative for asthenoteratozoospermia-related male infertility.

**Methods:**

Two related patients with asthenoteratozoospermia from a large consanguineous family were sequenced by whole-exome sequencing and Sanger sequencing to identify disease-causing genes. Scanning and transmission electron microscopy analysis revealed ultrastructural abnormalities of spermatozoa. Quantitative real-time PCR (qRT-PCR) analysis and immunofluorescence (IF) analysis were used to assess the expression of the mutant messenger RNA (mRNA) and protein.

**Results:**

A novel homozygous frameshift mutation (c.2823dupT, p.Val942Cysfs*21) in *DNAH6* was identified in both affected individuals and was predicted to be pathogenic. Papanicolaou staining and electron microscopy revealed multiple morphological and ultrastructural abnormalities of affected spermatozoa. qRT-PCR and IF analysis showed abnormal expression of DNAH6 in affected sperm, probably due to premature termination code and decay of abnormal 3′ untranslated region (UTR) region of mRNA. Furthermore, intracytoplasmic sperm injection could achieve successful fertilization in infertile men with *DNAH6* mutations.

**Discussion:**

The novel frameshift mutation identified in DNAH6 may contribute to asthenoteratozoospermia. These findings expand the spectrum of genetic mutations and phenotypes associated with asthenoteratozoospermia and may be useful for genetic and reproductive counseling in male infertility.

## Introduction

Infertility is a challenging health and social problem that affects 10%–15% of couples worldwide (1). A male factor is implicated in approximately half of infertility cases (2). Asthenoteratozoospermia, which describes sperm with reduced motility and obvious morphological abnormalities, is one of the most common factors in male infertility (3). Genetic causes of asthenoteratozoospermia include primary ciliary dyskinesia (PCD) and multiple morphological abnormalities of the sperm flagella (MMAF). PCD (mendelian inheritance in man (MIM): 244400) is characterized by dysfunction of motile cilia and flagella, leading to chronic rhinosinusitis, bronchiectasis, and heterotaxis (4), whereas MMAF is characterized by various morphological abnormalities of flagella (absent, short, bent, coiled, and irregular) (5). To date, 22 genes related to spermatogenesis or ciliogenesis have been identified as causative for MMAF, resulting in primary infertility without PCD (6–10). There is, therefore, a considerable genetic heterogeneity underlying asthenoteratozoospermia, not all of which has been accounted for.

Dynein axonemal heavy chain 6 (*DNAH6*; MIM: 603336), a member of the dynein protein family, is located at 2p11.2 in humans. *DNAH6* contains 77 exons and encodes a 4,158–amino acid protein (11, 12), and it plays an important role in multiple microtubule-associated motor protein complexes involved in ciliary and flagellar morphology and motility (13). Flagella and cilia are hair-like, microtubule-based structures with the same axoneme, formed by an ordered 9 + 2 arrangement of nine doublets of microtubules (A and B) and a central pair of microtubules (14). Dynein is arranged in complex arrays of single-headed, heterodimeric, and heterotrimeric outer dynein arm (ODA) and inner dynein arm (IDA) complexes (15). DNAH6 is a putative IDA and is required for motile cilia function (16). Several *DNAH6* mutations are known to cause severe sperm motility disorders and dysplasia of the fibrous sheath–MMAF (16, 17). *DNAH6* mutations may be related to MMAF in the absence of other PCD symptoms (18) but have recently been identified in patients with respiratory cilia disease leading to the evolution of PCD (16). *DNAH6* variants have also been implicated in lung function changes in patients with cystic fibrosis (19). In humans, loss-of-function mutations in *DNAH6* have been implicated in male infertility, such as globozoospermia, acephalic spermatozoa syndrome and azoospermia, and even premature ovarian insufficiency (20–22). However, the full spectrum of causative mutations in *DNAH6* is unknown, and there are a few reports of pathogenic *DNAH6* variations in Chinese populations.

Here, we identified a novel homozygous mutation in *DNAH6* in two infertile men with MMAF. We applied whole-exome sequencing (WES) and Sanger sequencing to a proband with idiopathic infertility and asthenoteratozoospermia from a consanguineous Uyghur family and subsequently identified the same homozygous mutation in his infertile brother. The brothers harboring the homozygous *DNAH6* mutation showed reduced progressive sperm motility, multiple morphological sperm malformations, and successful fertilization with intracytoplasmic sperm injection (ICSI) treatment. Our study adds to the *DNAH6* mutations reported in different patients with different diseases (**Table S1**), and the identification of this *DNAH6* mutation paves the way for a biomarker of asthenoteratozoospermia.

## Materials and methods

### Study participants

The proband (family member IV-1, 28 years old) and his family members in a consanguineous Chinese Uyghur family (Xinjiang, China) were recruited from the Reproductive Medicine Center in the Obstetrics and Gynecology Department of The Second Xiangya Hospital of Central South University for genetic counseling for primary infertility treatment. He was diagnosed with infertility 9 years before our study, and the initial physical and andrological examinations showed a normal body mass index and excluded associated diseases, such as hypogonadotropic hypogonadism, cryptorchidism, varicocele, seminal ductal obstruction, testicular trauma, and andrological tumor. The proband’s parents (III-1 and III-2) were first-degree cousins. The patient’s brother (IV-3) was also infertile for 4 years, and both wives had no fertility-related disorders (**Table S2**). Written informed consent was obtained from the study participants, and the study was approved by the Ethics Committee of The Second Xiangya Hospital of Central South University (Changsha, China).

### Semen and sperm morphology analysis

Semen samples were collected from the affected patients through masturbation after 3–5 days of sexual abstinence. Three repeated semen analyses were performed according to the World Health Organization (WHO, 2021) guidelines (23). Papanicolaou staining was used to assess sperm morphology, and morphological abnormalities of the flagella were classified as absent, short, coiled, bent, or irregular. For each subject, the percentages of morphologically normal and abnormal spermatozoa were documented according to the WHO guidelines.

### Transmission electron microscopy

Spermatozoa from the affected individual were fixed in 2.5% glutaraldehyde (Sigma-Aldrich) overnight at 4°C. Samples were immersed in 1% osmium tetroxide (OsO_4_, Taab), dehydrated in graded ethanol, and embedded in Taab812, dodecenylsuccinic anhydride, methylnadic anhydride, and dimethylaminomethyl phenol (Taab). Ultrathin 70- to 90-nm sections were contrast-stained with uranyl acetate and lead citrate, and we examined them by using a Tecnai G2 Spirit TWIN transmission electron microscope (FEI, USA) with a Gatan Orius CCD camera system.

### WES and Sanger sequencing

Peripheral blood samples were collected from family members (III-2, IV-1, IV-3, IV-5, and IV-9). Genomic DNA samples were extracted from peripheral blood using the QIAamp DNA Blood Mini Kit (QIAGEN). The patients (IV-1 and IV-3) underwent WES using the BGI Genomics platform (BGI-Shenzhen, China). The Agilent SureSelect Human All Exon v6 Kit (Agilent) was used to capture known exons and exon–intron boundary sequences (24, 25). Functional annotation and further filtering was performed using the 1000 Genomes Project, gnomAD, the database of single-nucleotide polymorphisms, and Exome Aggregation Consortium based on ANNOVAR. Potential pathogenicity was predicted *in silico* using SIFT, MutationTaster, PROVEAN, Polyphen-2, and CADD. Candidate variants were identified as previously described (26): (i) allele frequency <1% in the 1000 Genomes Project and gnomAD database as above; (ii) nonsynonymous or splicing variants, or coding INDELs; and (iii) *in silico* predicted to be pathogenic. The detailed variant interpretation and analysis pipeline were schematically presented previously (27, 28). Sanger sequencing was used to test family members and control subjects for the candidate pathogenic gene variants identified in the proband. Sanger sequencing was performed as a confirmatory test using the ABI 3730XL automated sequencer (Applied Biosystems, USA) according to the manufacturer’s instructions. The PCR primers are listed in **Table S3** and were synthesized by Tsingke Biotechnology Co., Ltd. (Beijing, China).

### Expression analysis

Total RNA was extracted from blood tissue of fertile adult donors who provided written informed consent for study participation and infertile patients using TRIzol reagent (Invitrogen). RNA (1 μg) was reverse-transcribed into cDNA using the HiScript III 1st Strand cDNA Synthesis Kit (+gDNA wiper) (Vazyme) according to the manufacturer’s instructions. We performed qPCR by using the ChamQ SYBR qPCR Master Mix (Vazyme) on a LightCycler96 real-time PCR product detection system (Roche, Switzerland) with specific primers of *DNAH6* mRNA (**Table S3**) to assess relative expression. Statistical analysis was performed using Student’s *t*-test in the Graphpad Prism 9.0 program (***p < 0.001). We also performed splicing analysis by using 2× Taq Plus Master Mix II (Vazyme) with specific primers listed in **Table S3**. The products were sequenced on an ABI 3730XL automated sequencer, and the sequencing results were analyzed using Chromas software (v2.6.5, Technelysium Pty Ltd., South Brisbane, Australia).

### Immunofluorescence analysis

Sperm samples were incubated with mouse monoclonal antibody targeting α-tubulin (T5168, Sigma-Aldrich; 1:1,000), rabbit polyclonal antibody targeting DNAH6 (ab122333, Abcam; 1:100), secondary antibodies [Alexa Fluor 488 anti-mouse immunoglobulin G (IgG) (A21121, Life Technologies; 1:1,000) and Alexa Fluor 555 anti-rabbit IgG (A31572, Life Technologies; 1:1,000)], and 4′,6-diamidino-2-phenylindole (DAPI). Fluorescence signals were captured using a BX-51 fluorescence microscope (Olympus, Japan). Images were analyzed using VideoTesT-FISH software (v.2.0, VideoTesT, St. Petersburg, Russia).

### *In vitro* fertilization and ICSI

*In vitro* fertilization (IVF) and ICSI procedures were performed as previously described (29). In brief, the female underwent a stimulation procedure and oocyte retrieval 35–36 h later after injection of human chorionic gonadotropin. Sperm samples collected from the patient were processed by conventional discontinuous density gradient centrifugation and swim-up procedures according to the WHO guidelines. For IVF, each oocyte was co-incubated with sperm to occur naturally. For ICSI, normal morphology spermatozoa were selected, immobilized, and injected into the oocyte cytoplasm. Then, the oocytes were cultured sequentially in cleavage embryo and blastocyst culture medium at 37°C under 5% CO_2_. Fertilization rates were evaluated on the morning of days 1–5 after oocyte retrieval.

## Results

### Clinical characteristics of the infertile patients

**Figure 1:**
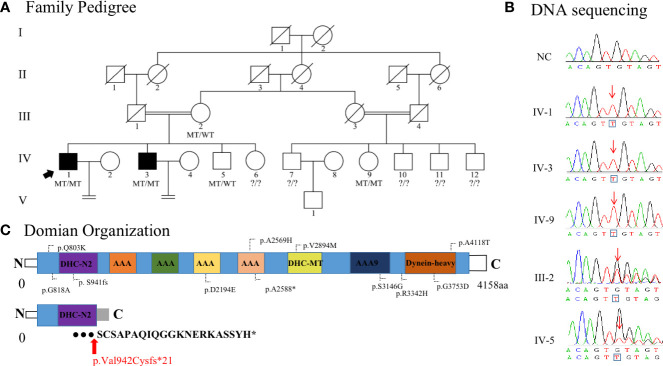
Identification of homozygous *DNAH6* variants in men with asthenoteratozoospermia. **(A)** Pedigrees of the Uyghur consanguineous families affected by homozygous *DNAH6* variants. Circles denote female family members, squares male family members, and solid symbols affected members. The double lines denote a consanguineous marriage, slashes denote deceased family members, and equal signs denote infertility. WT indicates wild type in the normal allele, MT indicates mutant type (c.2823dupT, p.Val942Cysfs*21) in *DNAH6*, and arrow indicates the index patient (family member IV-1). **(B)** Sanger sequencing results are shown on the right side. The variant positions are indicated by red arrows. **(C)** Structure of DNAH6 protein; predicted functional domains are shown together with the position of novel and old mutations identified in patients with asthenoteratozoospermia. The novel variant positions are indicated by red arrows. Expression analysis of DNAH6 mRNA and protein in the spermatozoa from a normal male control and men harboring homozygous *DNAH6* variants.

In the present study, we recruited a consanguineous Uyghur family consisting of two patients (IV-1 and IV-3) that were suffering from primary infertility (**Figure 1A**). The two patients were diagnosed with primary infertility, and both wives excluded fertility-related disorders. No apparent organic anomalies were found in the male reproductive system and the respiratory system by physical and andrological examination. Physical examination of the men revealed normal testicular size, external genital development, and bilateral spermatic veins. There was no history of respiratory disorders, situs inversus, exposure to hazardous environments, or drug/alcohol abuse in either man. Semen analysis showed adequate sperm concentration but abnormal motility, diagnosed as asthenoteratozoospermia, and sperm motility was significantly lower in IV-3 than in IV-1 (e.g., progressive motility, 2.4% vs. 21.3%) (**Table 1**). In the two patients (IV-1 and IV-3), the serum follicle-stimulating hormone, luteinizing hormone, and testosterone were within normal ranges, their karyotype were 46,XY, and no azoospermia factor microdeletion was found. The male cousin (IV-7) was the only IV generation male with a biologic offspring. The female member (IV-9), aged 20 years, was currently clinically normal (**Table S2**), with a normal menstrual cycle, normal development of the female reproductive system, and no history of respiratory disease. The pattern of inheritance of male infertility in the consanguineous families is consistent with an autosomal recessive homozygous mutation in the affected brothers.

**Table 1:** Semen characteristics and sperm morphology in men harboring homozygous deleterious *DNAH6* variants.

Individual		Semen (ml)	Concentration (10^6^/ml)	Number of motility sperm	Progressive motility (%)	Motility (%)
IV-1		2.5	48.3	525	21.3	42.9
IV-3		2	21.8	40	2.4	7.9
Reference limits		1.5 ^a^	15.0 ^a^	3.0 ^a^	32.0 ^a^	40.0 ^a^

Individual	Absent flagella (%)	Short flagella (%)	Coiled flagella (%)	Angulation (%)	Irregular caliber (%)	Abnormal head and neck (%)
IV-1	6	39	18	7	2.2	2
IV-3	8	45	27	8	3	3
Reference limits	–	–	–	–	–	–

a Reference limits according to the WHO standards.

### Sperm morphology by light and electron microscopy

**Figure 2:**
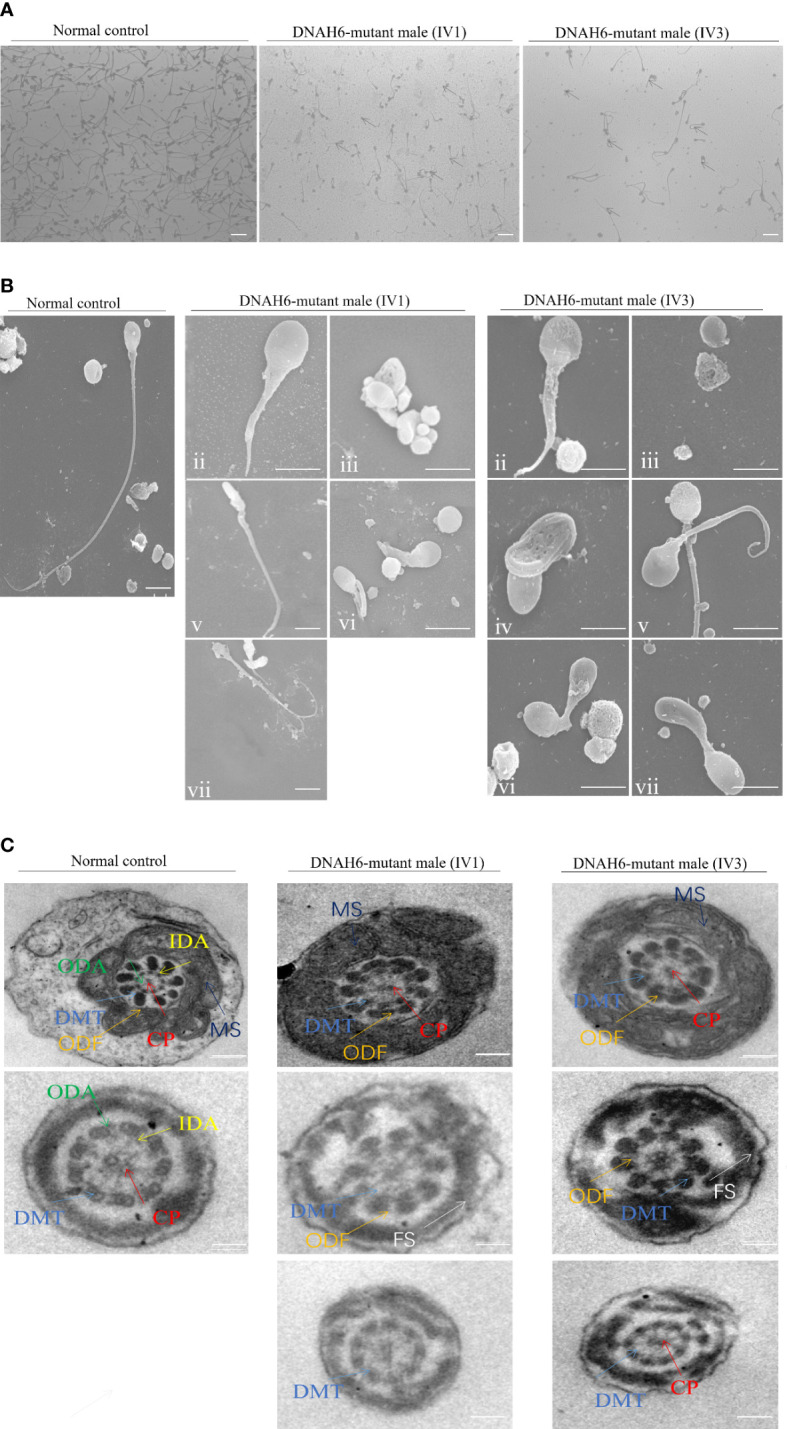
The phenotype of spermatozoa from the patient and a healthy control. **(A)** Staining for the spermatozoa obtained from a fertile control individual (NC) and men harboring *DNAH6* variants. Compared to the spermatozoa of NC, which presented long, smooth tails, most spermatozoa obtained from men harboring *DNAH6* variants displayed typical MMAF phenotypes, such as short, absent, coiled, and irregular flagella. Scale bars, 5 mm. **(B)** SEM analysis of the spermatozoa obtained from a fertile control individual (NC) and men harboring homozygous *DNAH6* variants. **(i)** Normal morphology of the spermatozoon from a healthy control male. (ii–vii) Most spermatozoa obtained from men harboring homozygous *DNAH6* variants displayed typical MMAF phenotypes, including short (ii), absent (iii), bent (iv), coiled (v), and irregular flagella (vi and vii). Scale bars, 5 μm. **(C)** Cross-sections of the midpiece and principal piece of the sperm flagella in the sperm obtained from NC displayed typical ‘‘9 + 2’’ microtubule structure and peri-axoneme structure. The axoneme microtubule structure, including nine pairs of peripheral doublet microtubules (DMT; indicated with white arrows) and the central pair of microtubules (CP; indicated with blue arrows), is visible. The ODAs (indicated with yellow arrows) and IDAs (indicated with red arrows) are also visible. The peri-axoneme structure includes a helical mitochondrial sheath (MS; indicated with green arrows), nine outer dense fibers (ODFs; indicated with orange arrows), and the fiber sheath (FS; indicated with pink arrows). Cross-sections of the midpiece, principal piece, and endpiece of the spermatozoa obtained from men harboring *DNAH6* variants revealed that typical axonemal anomalies were disorder, including partial or complete absence of peripheral doublet microtubules and the central pair of microtubules and dynein arms, whereas other axoneme microtubule structures seemed to be unaffected. Scale bars, 200 nm.

To investigate sperm morphology and flagellar ultrastructural abnormalities in the two men harboring the *DNAH6* variant, we performed Papanicolaou staining, microscopic examination, and scanning and transmission electron microscopy (SEM and TEM, respectively) analyses of the sperm from these individuals (IV-1 and IV-3). Papanicolaou staining and SEM showed that most spermatozoa had abnormal tails, including short, coiled, bent, occasionally absent flagella, and irregular flagella compared to unaffected controls (**Figures 2A, B**). TEM revealed ultrastructural disruption of affected spermatids, with disordered peripheral dense fibers, serious distortions in axonemes, including sporadic to partial or complete absence of the central complex (9 + 1 or 9 + 0), and frequent absence of outer doublet microtubules (DMT; 8 + 0, 8 + 2). Dynein arms (inner or both) were frequently absent from the axoneme peripheral doublets, and the axoneme was sometimes completely disrupted. Spermatozoa from healthy controls showed the typical “9 + 2” axoneme microtubule structure (**Figure 2C**).

### Identification of the *DNAH6* variant

We performed WES to identify a potential genetic basis for asthenoteratozoospermia in the proband and his brother. WES produced 126.34 million clean reads and 116.27 million total effective reads, with 99.91% of the reads mapping to the human reference genome. The mean sequencing depth on target regions was 120.93, and 98.98% of target regions were covered 10× or more. A total of 98,802 single-nucleotide polymorphisms and 14,863 indels were detected in the proband. As the patients were from a consanguineous family, homozygous variants implicated in infertility phenotypes were prioritized. After screening, only one novel homozygous frameshift variant (NM_001370.2: exon 17: c.2823dupT, p.Val942Cysfs*21) in *DNAH6*, known to cause asthenozoospermia, fulfilled these criteria. The record of this variant in public databases (ClinVar, gnomAD, and 1000 Genomes) showed in **Table 2**. *In silico* functional prediction classified the c.2823dupT variant as deleterious with SIFT, MutationTaster (a score near or equal to 1), PROVEAN (score of −9.06), PolyPhen-2 (score of 1.000, specificity of 1.00, and sensitivity of 0.00), and CADD (score of 19.6) (**Table 2**). The *DNAH6* homozygous variants in the affected individuals (IV-1 and IV-3) and female member (IV-9) and the *DNAH6* heterozygous variants in the proband’s brother (IV-5) and mother (III-2) were further confirmed by Sanger sequencing (**Figure 1B**), consistent with autosomal recessive inheritance. The frameshift variant was not detected in a sample of 300 Chinese male controls.

**Table 2:** Homozygous deleterious *DNAH6* variants identified in men with asthenoteratozoospermia.

variant ^a^	Amino acid change	Zygosity	1000 Genomes Project	East Asians in gnomAD	All individuals in gnomAD	SIFT	Mutation Taster	PROVEAN^b^	PolyPhen-2	CADD^c^
c.2823dupT	p.Val942Cysfs*21	Homozygous	0	0	0.00003428	D	D	−9.06 (D)	1.000 (D)	19.6 (D)

D damage.

a The NCBI reference sequence number of DNAH6 is NM 001370.2.

b Variants with scores lower than −2.5 (cutoff) are predicted to be deleterious.

c Variants with CADD values greater than 4 are considered to be deleterious.

### Effect and expression of the *DNAH6* variant

Evolutionary conservation analysis showed that valine 942 in human DNAH6 is highly conserved between species, suggesting that this site is functionally important. The frameshift variant was located in key functional domains of the coding protein and was predicted to result in a premature termination codon (PTC) at site 961 (**Figure 1C**).

**Figure 3:**
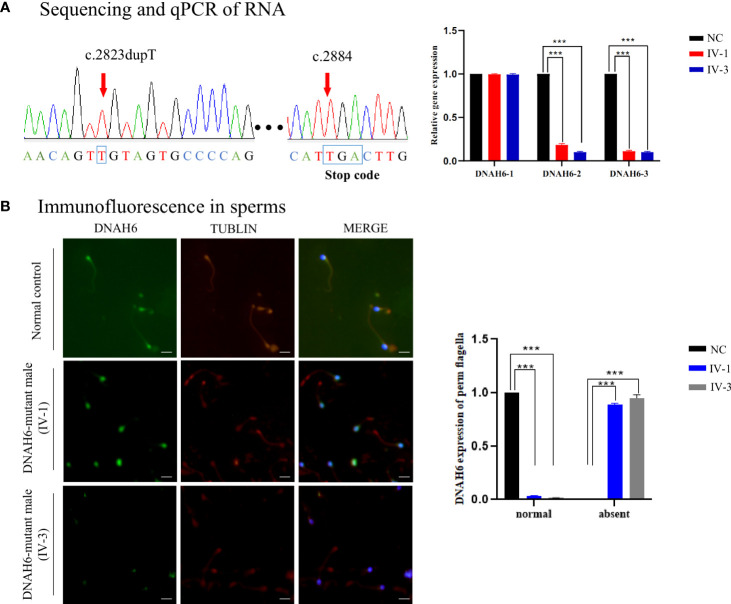
DNAH6 expression in men with asthenoteratozoospermia. **(A)** Real-time quantitative PCR analysis indicated that the abundance of homozygous DNAH6 mRNA was significantly reduced in the sperm from men harboring *DNAH6* variants and sequence analysis that it happened to terminate at the PTC position, when compared to that of a normal control man. Data represent the means + standard error of measurement of three independent experiments. Two-tailed Student’s paired or unpaired t-tests were used as appropriate (***p < 0.001). **(B)** Representative images of spermatozoa obtained from a fertile control individual (NC) and men harboring *DNAH6* variants (IV-1 and IV-3) stained with the anti-DNAH6 antibody, anti–a-tubulin antibody, and DAPI. Staining results revealed that DNAH6 was localized in the neck and almost reduced in the sperm flagella obtained from men harboring *DNAH6* variants but localized in the neck and tail of the sperm obtained from the NC. Representative data are provided to illustrate the typical staining observed in *DNAH6*-associated cases. Scale bars, 5 mm.

To explore the impact of the *DNAH6* p.Val942Cysfs*21 variant, we performed immunostaining (IF) and qPCR assays to detect the DNAH6 protein and mRNA levels in the two patients. IF showed that DNAH6 protein was located along the neck and along the entire length of the sperm flagella in a control individual, but only in the neck of mutant sperm (**Figure 3B**). In addition, qRT-PCR analysis of nonsense-mediated mRNA decay demonstrated that DNAH6 happened to terminate at the PTC position and to decay of abnormal 3′ UTR region of the mRNA (**Figure 3A**), whereas splicing analysis showed no alternative splicing (**Figure S1**).

### *In vitro* fertilization, intracytoplasmic sperm injection, and pregnancy outcome

The proband couple underwent two cycles at our clinic in 2020. In the first IVF cycle, 15 oocytes were received and only one good-quality embryo was available. Unfortunately, this resulted in an early unexplained miscarriage after transfer. In the second half-ICSI cycle, 13 oocytes were obtained, of which six were fertilized with IVF and seven with ICSI. Only one oocyte was fertilized by IVF, and no good-quality embryo was obtained. Meanwhile, six in the seven oocytes were successfully fertilized by ICSI, whereas one was fertilized abnormally. Finally, three good-quality embryos were formed, suggesting that spermatozoa harboring *DNAH6* mutations can produce viable embryos with ICSI treatment. However, the proband’s wife did not become pregnant after transfer (**Table 3**).

**Table 3:** Clinical outcomes of IVF/ICSI treatment cycles using the for spermatozoa from men harboring homozygous DNAH6 variants.

Patient	Female age (years)	Male age (years)	Duration of infertility (years)	Cycles	Insemination method	Total no. of oocytes	Total fertilization rate (%)	Normal fertilization rate (%)	Good-quality embryo rate (%)	No. of live births (n)
IV-1	25	27	9	1	IVF	15	4/15 (26.7)	2/15 (13.3)	1/15 (6.7)	0
				2	IVF	6	1/6	0	0	0
					ICSI	7	7/7 (100)	6/7 (85.7)	3/7 (42.9)	0
IV-3	23	24	5	NA	–	–	–	–	–	0

## Discussion

Asthenoteratozoospermia describes the absence or reduction of motile sperm in the ejaculate, and it is a known cause of male infertility (~19% of infertile men) (1, 30). However, the causes of asthenoteratozoospermia remain unclear. Here, we discovered and validated a novel homozygous frameshift mutation p.Val942Cysfs*21 in *DNAH6* by both WES and Sanger sequencing. This *DNAH6* variant is absent in population analyses and was predicted to be damaging by several *in silico* analyses. The homozygous *DNAH6* p.Val942Cysfs*21 mutation results in a typical asthenoteratozoospermia phenotype, with no obvious phenotypic differences between our patients and other previously reported cases of asthenoteratozoospermia, regardless of the mutations involved (31).

Note that the homozygous mutation in *DNAH6* was identified in this family. The male family members (IV-10, IV-11, and IV-12) are suggested to examine the *DNAH6* p.Val942Cysfs*21 mutation, which is helpful for their choice of childbearing methods. The single female family member IV-9 is homozygous for the mutation and is unmarried, whose reproductive outcome needs to be followed. There were a lot of severe abnormal morphologies and ultrastructural disruptions in the affected sperm. Pathogenicity analyses showed that DNAH6 was abnormally expressed in spermatozoa from the men harboring the variant. In contrast to the DNAH6 protein in the neck and the entire length of normal control sperm and the absence of DNAH6 staining in the spermatozoa of other previously reported patients (32), the mutant DNAH6 was only present in the neck of our patient’s sperm. We propose that the difference could be explained by the instability of the structure of the truncated protein. The observation of residual mRNA expression and decay of the mRNA 3′ UTR tail of the mutant *DNAH6* gene supports our hypothesis.

Dynein is a component of microtubule-associated motor protein complexes and plays an important role in cilial and flagellar motility or in the cytoplasm, where it mediates intercellular movement and cytoskeletal remodeling (13). DNAH6 is mainly expressed in the human testis and is needed for motile cilia function (16, 21). Mutations in *DNAH6* have been associated with PCD and heterotaxy caused by the central pair complex motile cilia dysfunction (16). Mutations in two IDA heavy-chain protein-encoding genes, *DNAH1* (MIM: 603332) and *DNAH2* (MIM: 603333), and two ODA heavy chain components, *DNAH8* (MIM: 603337) and *DNAH17* (MIM: 610063), have been described in individuals with isolated male infertility due to asthenoteratozoospermia. *DNAH1* is required for the formation of IDAs in spermatozoa and is important for the assembly and biogenesis of the flagellar axoneme, and mutations in *DNAH1* cause MMAF and other PCD-associated symptoms (33). *DNAH6* can act both recessively and possibly through trans-heterozygous interactions with other PCD genes such as *DNAH1* or *DNAH5*. Dynein is involved in spermatogenesis, and other patients with a rare homozygous missense mutation in *DNAH6* exhibited azoospermia and oligozoospermia and male infertility (21, 34). *DNAH6* deficiency is also associated with asthenoteratozoospermia in the absence of other PCD symptoms (18). Our phenotypic analysis revealed that the men carrying the *DNAH6* variant displayed typical MMAF phenotypes, including reduced sperm motility and MMAF, without other PCD symptoms. TEM analysis further revealed partial defects or loss of the dynein arms and severe disorganization or aberration of axonemal or other peri-axonemal microtubule structures in spermatozoa. Therefore, DNAH6 is beneficial for flagellar axoneme assembly during spermatogenesis, and the asthenoteratozoospermia-associated phenotypes in these cases are likely to be caused by homozygous variants in *DNAH6*.

From the clinical perspective, ICSI is an assisted reproductive technology that is an effective method to achieve successful conception in infertile men with MMAF (35–39). Previous studies have reported success with ICSI in a series of individuals with MMAF-related gene mutations. For example, MMAF-affected individuals with biallelic variants in *DNAH1*, *DNAH8*, or *TTC29* have good clinical outcomes following ICSI (40–42), whereas failed pregnancies have been reported in MMAF-affected men with *CEP135* (MIM: 611423), *DNAH17*, or *CFAP65* variants (21, 43, 44). It has also been reported that patients with MMAF with *DNAH6* mutations do not achieve pregnancy (18). In our study, fertilization was also achieved with ICSI (7 of 7, 100%), whereas 23.8% (5 of 21) with IVF. However, whether successful ICSI outcomes can be achieved in patients with MMAF with *DNAH6* mutations requires further investigation.

## Conclusion

In conclusion, our experimental observations in human subjects show that a novel frameshift mutation in *DNAH6* can induce MMAF-associated asthenoteratozoospermia. This finding expands the spectrum of genetic mutations associated with MMAF and asthenoteratozoospermia. Additional functional analysis of specific *DNAH6* mutations would provide further important information about the underlying genetic causes of male infertility, allowing genetic counselors and clinicians to develop personalized treatment plans.

## Data availability statement

The primary data for this study are available from the corresponding author upon reasonable request.

## Ethics statement

The studies involving human participants were reviewed and approved by the Institutional Review Board from the Ethics Committee of the Second Xiangya Hospital, Central South University. The patients/participants provided their written informed consent to participate in this study.

## Author contributions

H-LH and WS: Data collection or management, data analysis, and manuscript revision process. FH and JuZ: Project development, data analysis, and manuscript editing. DL and JiZ: Data analysis and manuscript editing. BL, JG, and RY: Manuscript editing and revision. XS and JC: Data collection or management, data analysis, and manuscript writing. All authors contributed to the article and approved the submitted version.

## Supplementary material

Supplementary Figure 1 Sanger sequencing results of splicing analysis in men harboring *DNAH6* variants. The variant positions are indicated by red arrows.
