# Supplementary material for: A novel frameshift mutation in DNAH6 associated with male infertility and asthenoteratozoospermia
Source: Front Endocrinol (Lausanne). 2023 Jun 22;14:1122004. doi: 10.3389/fendo.2023.1122004 (PMC10324608; doi:10.3389/fendo.2023.1122004)
Supplement: Supplementary file 1 [file Table_1.docx]

**Table S1** Summary of reported *DNAH6* mutations.

| Identified genes | Nucleotide change | Amino acid change | Mutation | Zygosity | Associated disease | References |
| --- | --- | --- | --- | --- | --- | --- |
| *DNAH6* | c.1316+1_1316+2insC | N/A | NA | Heterozygote | Severe sperm motility  Disorders, DFS-MMAF | 16, 17 (2016,2021) |
|  | c.7762C>T | p.Arg2588* | Nonsense | Heterozygote | Severe sperm motility  Disorders, DFS-MMAF |  |
|  | NA | NA | NA | NA | azoospermia | 22 (2017) |
|  | Rs 1192269(G>C) |  | Missense |  | Cystic Fibrosis in Lung | 19 (2018) |
|  | Rs 28375417(G>A) |  | Missense |  | Cystic Fibrosis in Lung |  |
|  | (C>A) |  |  |  | Cystic Fibrosis in Lung |  |
|  | c.2454A>T | p.Glu818Asp | Missense | Compound heterozygous | Globozoospermia and acephalic spermatozoa | 20 (2018) |
|  | c.7706G>A | p.Arg2569His | Missense |  |  |  |
|  | c.6582C>A | p.Asp2194Glu | Missense | Compound heterozygous | MMAF | 18 (2019) |
|  | c.11258G>A | p.Gly3753Asp | Missense |  |  |  |
|  | c.2823dupT | p.Val942Cysfs*21 | Missense | Compound heterozygous | MMAF |  |
|  | c.10025G>A | p.Arg3342His | Missense |  |  |  |
|  | c.2407C>A | p.Gln803Lys | Missense | Compound heterozygous | POI | 22 (2020) |
|  | c.8680G>A | p.Val2894Met | Missense |  |  |  |

**Table S2** Basal hormone levels in women.

| Individual | FSH (mIU/ml) | LH (IU/ml) | PRL (ng/ml) | E_2_ (pmol/l) | P (ng/ml) | TSTO (ng/dl) |
| --- | --- | --- | --- | --- | --- | --- |
| IV-2 | 7.22 | 4.99 | 26.12 | 43.51 | 0.29 | 29.18 |
| IV-4 | 4.71 | 5.54 | 13.65 | 51.24 | 0.20 | 31.61 |
| IV-9 | 4.56 | 3.77 | 17.56 | 40.64 | 0.24 | 34.42 |
| Reference limits | 3.03-8.08 | 2.39-6.60 | 5.18-26.53 | 21-251 | 0.1-0.3 | 12.33-59.48 |

**Table S3** PCR primers of *DNAH6*.

| Type | Name | Primer sequence |
| --- | --- | --- |
| Sanger sequencing |  | F: 5’-CCAAATTTGATTGCCTGGAT-3′  R: 5′-CCCTTACCACTTGGGAAACA-3′ |
| nonsense-mediated mRNA decay | DNAH6-1 | F: 5′-AGGAGAAGACAAGAAAACGAC-3′  R: 5′-TTGCTATGCTTACTGGACG-3′ |
|  | DNAH6-2 | F: 5′-AGAAGTCCTAAACGGTCAAG-3′  R: 5′-GGCATCAACTGTTTGTTCAA-3′ |
|  | DNAH6-3 | F: 5′-CTATAAGCCAAGCCCAACA-3′  R: 5′-GAGCAAAGCTGATCCCTT-3′ |
|  | GAPDH | F: 5′-GGGGCTCTCCAGAACATC-3′  R: 5′-TGACACGTTGGCAGTGG-3′ |
| alternative splicing |  | F: 5′-TCAAACAATTGCTCTGGGATT-3′  R: 5′-TTGTTTTTGCCATTCATCCA-3′ |
