# Supplementary figures and images for: A novel frameshift mutation in DNAH6 associated with male infertility and asthenoteratozoospermia
Source: Front Endocrinol (Lausanne). 2023 Jun 22;14:1122004. doi: 10.3389/fendo.2023.1122004 (PMC10324608; doi:10.3389/fendo.2023.1122004)

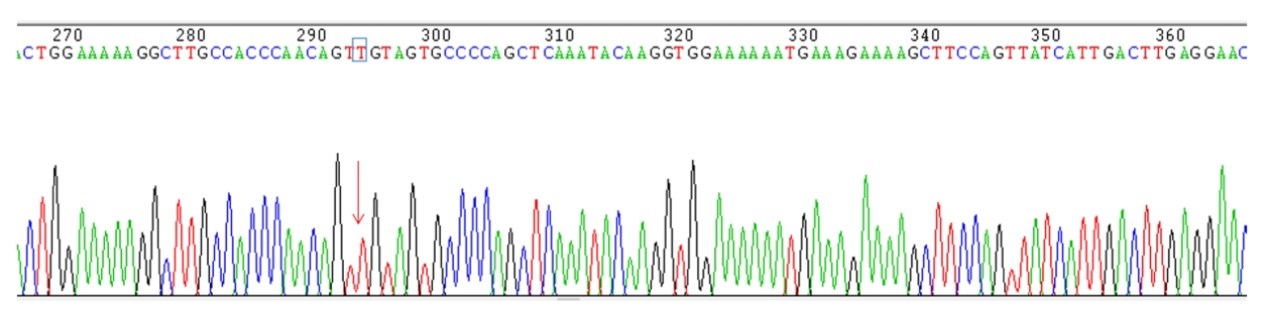

Supplement: Supplementary Figure 1 — Sanger sequencing results of splicing analysis in men harboring DNAH6 variants. The variant positions are indicated by red arrows. [file Image_1.png]
